# Progress Veterinary Science

**Published:** 1886-04

**Authors:** 


### Progress of Veterinary Science.

VETERINARY MEDICINE IN PRUSSIA.—The annual appropriation for the expenses of the veterinary school, and the salaries of the veterinary police in Prussia for the year 1886 is $189,333.93.

COCAINE POISONING IN A CAT.—Dr. H. J. Boldt presented, at a meeting of the New York Pathological Society, the brain and medulla of a cat which had died in convulsions twelve minutes after an injection of eleven minims of a thirty-three-and-a-third-per-cent. solution of cocaine. The first effect noticed was a sort of leaping gait with dragging of the hind extremities, followed by convulsions. There was simulation of the Cheyne-Stokes respiration. The temperature in the rectum after death was 104° F. At the autopsy, made half an hour after death, the vessels of the pia mater in both brain and cord were much congested, there was an extravasation of blood in the fourth ventricle, also a small amount in the anterior part of the medulla, and there were minute extravasations throughout the brain. The lungs were collapsed, and contained no blood; the right ventricle of the heart was overdistended, the left one empty. There was congestion of the gray substance of the cord. Dr. Boldt said that death seemed to have taken place from paralysis of the respiratory center.

Rectum of the Armadillo.—We have been struck in the case of two armadillos we recently dissected, with the presence of an enormous dilatation of the rectum filled with earth. In one case, this dilatation certainly contained eight cubic inches of earth. Does the armadillo swallow the earth with its food, and it then accumulates thus, or does a paralysis of the sphincter occur in captivity, and the earth force its way in? It scarcely seems as if such a state of affairs could be physiological, and it must be a source of discomfort, if not of danger, for in the one instance the empty *pupa* of the carrion-fly were found in this earth in large numbers. ***

Immunity from Contagious Diseases.—Drs. D. E. Salmon and T. B. Smith have just published ("Proc. Biol. Soc. of Washington," Vol. III. a remarkable discovery made by them of a new method of producing immunity from contagious diseases. By experimenting upon pigeons, they were able to establish an immunity from the disease known as swine-plague by the inoculation of solutions, in which pathogenic bacteria had been cultivated, and afterwards destroyed by heat. The conclusions they reached are as follows: Immunity is the result of exposure bioplasm of the animal body to the chemical products of the growth of the specific microbes which constitute the virus of contagious fevers; 2d. These particular chemical products are produced by the growth of the microbes in suitable culture-liquids in the laboratory, as well as in the liquids and tissues of the body; 3d. Immunity may be produced by introducing into the animal body such chemical products as have been produced in the laboratory.—*Science.*

